# Flexural and Shear Strain Characteristics of Carbon Fiber Reinforced Polymer Composite Adhered to a Concrete Surface

**DOI:** 10.3390/ma11122596

**Published:** 2018-12-19

**Authors:** Hisham Jahangir Qureshi, Muhammad Umair Saleem

**Affiliations:** Department of Civil and Environmental Engineering, College of Engineering, King Faisal University, 31982 Hofuf, Alahsa, Saudi Arabia; hqureshi@kfu.edu.sa

**Keywords:** carbon fiber reinforced polymer composites, debonding, tensile strain, shear strain, epoxy, concrete interface

## Abstract

The use of Fiber Reinforced Polymer (FRP) composites for strengthening concrete structures has gained a lot of popularity in the past couple of decades. The major issue in the retrofitting of concrete structures with FRP is the accurate evaluation of flexural and shear strains of polymer composites at the bonding interface of epoxy and concrete. To address it, a comprehensive experimental study was planned and carbon fiber reinforced polymer (CFRP) composite was applied on the concrete surface with the help of adhesives. CFRP was used as an external mounted flexural and shear reinforcement to strengthen the beams. Flexural load tests were performed on a group of eight reinforced concrete beams. These beams were strengthened in flexural and shear by different reinforcement ratios of CFRP. The strain gauges were applied on the surface of concrete and CFRP strips to assess the strain of both CFRP and concrete under flexural and shear stresses. The resulting test data is presented in the form of load–deformation and strain values. It was found that the values of strains transferred to the FRP through the concrete are highly dependent on the surface tensile properties of concrete and debonding strength of the adhesive. The test results clearly indicated that the strength increment in flexural members is highly dependent on strain values of the CFRP.

## 1. Introduction

Reinforced concrete is one of the most widely used construction materials in the past, as well as in the modern world, due to its high compressive strength, fire and weather resistance properties. The majority of reinforced concrete structures that exist nowadays were designed and constructed based on old design codes and specifications. Most of these concrete structures are more than fifty years old, and during their design life, with the passage of time, they are subjected to harsh environmental conditions, e.g., saltwater, deicing chemicals, and extreme temperature variations, hence resulting in strength reduction. Therefore, there is a need to strengthen and save old existing infrastructures by utilizing various strengthening techniques. 

In the past, the most widely used method, which was not very cost economical and was adopted by professional engineers, was to demolish and reconstruct the old civil infrastructure or to add an extra member in order to increase the load-carrying capacity of the existing structure. One of the strengthening techniques adopted by professional engineers recently to strengthen and increase the service life of reinforced concrete structures is to use FRP, also known as fiber reinforced plastic. The use of externally bonded Carbon Fiber Reinforced Polymer (CFRP) has emerged as an efficient solution for the structural strengthening of structures. CFRP has shown outstanding results when employed for rehabilitation and strengthening of concrete structures. Various types of cracks (flexural, flexural shear and shear) do appear during the lifespan of the structures, which can be prevented with the use of CFRP composite. CFRP fibers resist fatigue and have shown good resistance against tensile loads. Moreover, CFRP has strong proven resistance against harsh environmental conditions such as corrosion, and has shown a near-zero coefficient of thermal expansion, which is known to provide longer life and fewer maintenance requirements [1,2]. Recently, many structures have been strengthened using CFRP materials. CFRP has a low weight-to-volume ratio, high stiffness-to-weight ratio, and flexibility, and has attracted many researchers’ interest around the world, who have investigated the effectiveness and feasibility of using CFRP materials to strengthen concrete and masonry structures [1,2,3,4,5,6,7,8,9,10]. 

CFRP bonded externally to the concrete surface results in an increase in the load-carrying capacity of concrete structural members by up to three to six times, as compared to the unreinforced structure [3,4]. However, when CFRP is applied to the concrete surface in the form of externally bonded flexural and shear strips, it results in debonding and sometimes premature failure of the CFRP concrete interface. This phenomenon becomes more critical at the mid-span and supports of a simply supported beam section [11,12,13,14,15]. Some of the major challenges faced by modern civil engineering are the strengthening of existing deteriorated concrete and masonry structures. The failure of CFRP-strengthened beams may occur due to flexural, shear, bearing, CFRP debonding, CFRP rupture and by the ripping of the concrete cover along with CFRP [16,17,18]. 

## 2. Literature Review 

The effective utilization of FRP attached to the concrete surface has remained an area of research worldwide. Over the last three decades, significant advancement has been observed in the use of externally bonded Fiber Reinforced Polymer (FRP) in the rehabilitation and strengthening of reinforced concrete structures. 

Kotynia [19] carried out experimental tests on reinforced concrete (RC) beams externally strengthened with CFRP strips in flexure and discussed the flexural behavior of the beams, as well as their failure modes. CFRP strain efficiency was also discussed in relation to different strengthening modes. The behavior of FRP repaired beams was determined analytically by Arduini and Nanni [20]. Piamanmas and Pornpongsaroj [21] conducted an experimental research program on the peeling behavior of reinforced concrete beams strengthened by three CFRP wrapping schemes under different end conditions. The behavior of bond between the FRP and the concrete is a key factor in controlling the failure modes/mechanisms of concrete structures strengthened with FRP composites. Yao et al. [22,23] conducted an experimental study on the bond shear strength between FRP and concrete by using a near-end supported (NES) single-shear pull test and presented insights on plate end debonding failures in reinforced concrete beams. Gao et al. [24] conducted research on the failure diagrams of FRP strengthened RC beams. They established a failure diagram which shows the relationship and the transfer tendency among different failure modes for reinforced concrete beams strengthened with FRP strips. Their established diagram also explains how the failure modes change with FRP thickness and the distance from the end of the FRP strips to the support.

Cao et al. focused their research on the distribution of strains in the FRP strips intersected by the critical shear crack, and the shear capacity at debonding failure [25]. A simple model was proposed in their research to predict the contribution of FRP to the shear capacity of the beam at the complete debonding of the critical FRP strip. Teng and Chen conducted research on the debonding failure of RC beams strengthened externally with FRP reinforcement [26]. Kang et al. conducted a review on past research programs in relation to debonding failures of FRP laminates externally attached to concrete [27]. Lee et al. conducted their research on the effective strain of RC beams strengthened in shear with FRP [28]. They presented the results of their analytical and experimental study in the performance of reinforced concrete beams strengthened in shear with FRP composites and internally reinforced with conventional steel stirrups. Li et al. performed experimental tests on concrete beams which were strengthened with externally bonded CFRP sheets to investigate debonding initiation and tensile strain of FRP laminates, and investigated the allowable tensile strain of FRP-strengthened RC beams in comparison to design code provisions [29]. Hasnat et al. enhanced the debonding strain limit for CFRP-strengthened RC Beams by using U-Clamps and tried to resist premature cover debonding failure [30]. Lopez et al. proposed a new interfacial fracture energy model by characterizing the FRP-concrete interface with beam-type tests [31].

Mostafa et al. predicted the debonding load for FRP strengthened reinforced concrete members and proposed a nonlinear analytical model to determine the interfacial shear and normal stresses at the FRP–concrete interface for reinforced concrete beams externally strengthened with CFRP sheets [32,33]. Chen et al. [34] presented an analytical study on the progressive failure of FRP wraps in strengthened beams. In their research study, the debonding and the subsequent rupture processes were derived, and the FRP contribution to the shear capacity of the beam was quantified. Later, the analytically proposed solution was verified by comparing its predictions with the predictions of a finite element model. Fu et al. presented the results of an experimental study into the effect of U-jacketing on delaying or suppressing debonding failure [35].

Abid and Lami [36] conducted an extensive review of past research works related to the strength and durability of concrete beams externally bonded with CFRP. A special focus was on the bond behavior, testing techniques and models used to assess bond strength. In addition, the deterioration of the FRP due to moisture was also examined. Flexural, shear and fatigue behaviors of different strengthening techniques were also reviewed in detail. 

While discussing the above-mentioned literature review, it was found that the effect of CFRP reinforcement ratio on flexural and shear strain at the CFRP–concrete bonding interface needs further exploration. In addition to this, the post-peak behavior and residual load-carrying capacity of the cracked CFRP-strengthened beams require further investigation. In contrast to the aforementioned literature survey, the current study is one of a kind, in which CFRP in the form of strips of varied widths was used as an external reinforcement to strengthen reinforced concrete beams against flexural cracks and shear. This article presents an experimental study which was conducted with the objectives of: (1) determining the effect of CFRP reinforcement ratio on the shear strain and flexural strain profiles of reinforced concrete when subjected to bending; and (2) understanding the effect of different CFRP reinforcement ratios and strengthening layouts on the failure behavior of RC beams. To investigate the effective flexural strain and shear strain of the CFRP strengthened beams, eight RC beams, including two control samples, were tested under four-point bending loads. These beams had different CFRP reinforcement layouts and exhibited two distinct modes of failures (flexural or shear), based upon the amount of externally applied CFRP reinforcement. Included in the study are the peak loads, deformations, post-peak behavior, failure modes and strains of concrete and CFRP at shear and flexural failure of reinforced concrete beams.

## 3. Experimental Plan

**Figure 1:**
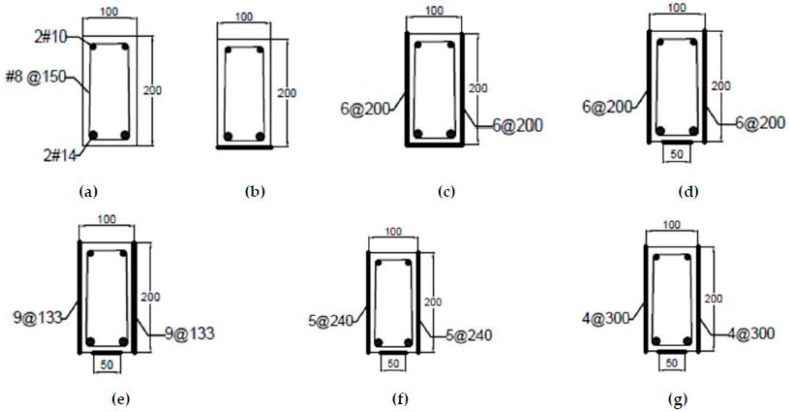
Cross-section details of test specimens: (**a**) Control samples, (**b**) B1 (F+0), (**c**) B2 (F+S), (**d**) B3 (F+S), (**e**) B4 (F+S), (**f**) B5 (F+S), and (**g**) B6 (F+S).

**Figure 2:**
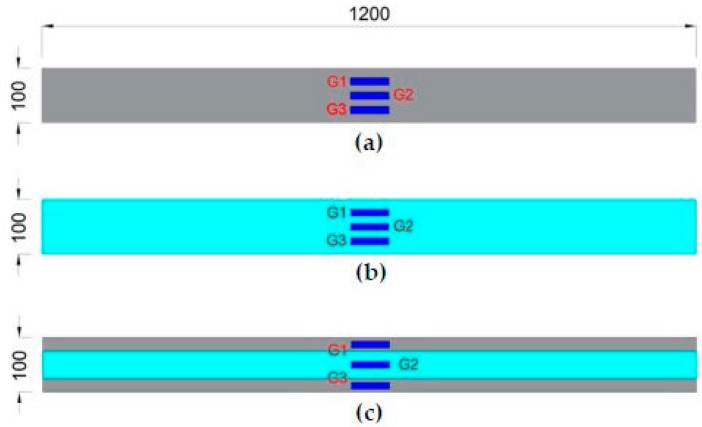
Position of strain gauges for flexure: (**a**) Control samples, (**b**) B1, B2 (**c**) B3, B4, B5 and B6.

**Figure 3:**
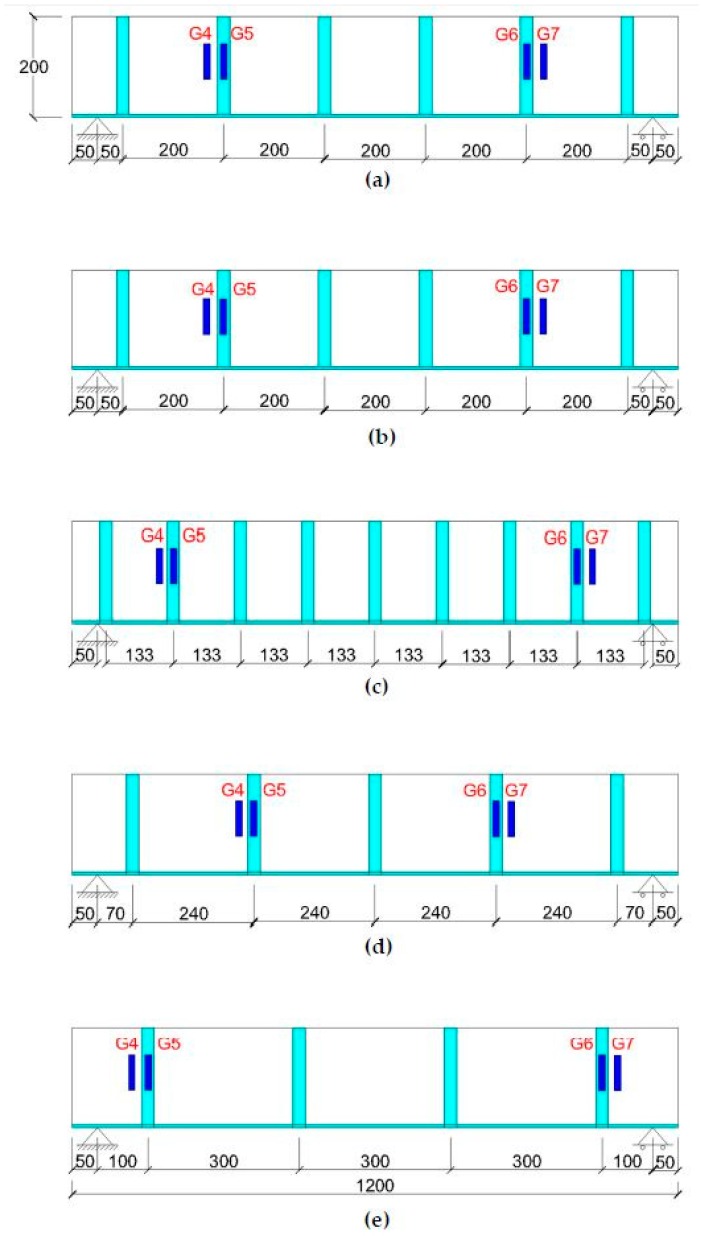
Position of strain gauges for shear: (**a**) B2, (**b**) B3, (**c**) B4, (**d**) B5 and (**e**) B6.

In this research work, eight reinforced concrete beams were prepared and tested for flexure and shear under a four-point flexural test setup, and the resulting strains values were measured at different locations through strain gauges (Tokyo Sokki, Tokyo, Japan) which were installed on the beam surface at the location of critical stresses. The beams were strengthened with different flexural and shear reinforcement ratios. Table 1 and Figure 1, Figure 2 and Figure 3 give the details of all beam specimens and their strengthening layout. All the reinforced concrete beams selected for tests had the same cross-sectional parameters (100 mm × 200 mm), as well as an equal overall length of 1200 mm. Two beams out of eight were selected as control samples, while the rest were strengthened with external CFRP strips of 1.5 mm thickness at different ratios in order to control flexural and shear cracks. For the purpose of comparison between different experimental beams, the internal reinforcement ratio, location of the strain gauges, cross-sectional sizes and overall length were kept constant. Details of all the parameters of the eight reinforced concrete beams are given in Table 1. Table 1 gives the flexural and shear CFRP reinforcement ratios. Reinforcement ratio at a particular section is calculated by dividing the cross-sectional area of CFRP with the cross-sectional area of the concrete beam. CFRP flexural reinforcement ratio (***ρ_f_***) corresponds to the quantity of CFRP which is applied at the bottom face of the beams. For instance, a CFRP strip width of 100 mm with a thickness of 1.5 mm will result in a CFRP flexural reinforcement ratio of 0.0075 (1.5 × 100 / (100 × 200) = 0.0075). However, the shear reinforcement ratio (***ρ_s_***) is provided in the form of CFRP strips, which are uniformly distributed on both side faces of the beams, as shown in Figure 1 and Figure 3. The total CFRP reinforcement ratio (***ρ_v_***) is the sum of flexural (***ρ_f_***) and shear (***ρ_s_***) CFRP reinforcement ratio. To understand the effect of CFRP flexural and shear reinforcement on the overall capacity of the beams, the flexural and shear CFRP reinforcement ratios are schematically varied. 

A thickness of 1.5 mm was kept constant in all CFRP strips applied externally to the beam surface. CFRP was applied over the surface of concrete beams by adopting the wet layup method. During the experimental work, CFRP strip width and reinforcement ratios were the two design parameters under study. Figure 1 shows the cross-section of the beams and their reinforcement details, whereas the location of the strain gauges is given in Figure 2 and Figure 3. Figure 2 shows the bottom view of the tested specimens with the location of the flexural strain gauges. Three strain gauges were connected at the bottom face of the beams to measure the flexural strain only. However, Figure 3 shows the locations of shear strips, along with the location of the strain gauges, which were used to measure the shear strains in the beams.

## 4. Material Properties

Reinforced concrete beam samples were prepared from concrete with a compression strength of f_c_′ = 28 MPa, and a deformed steel flexural reinforcement comprising 14 mm nominal diameter rebars having f_y_ = 420 MPa was used as internal reinforcement, as shown in Figure 1a. To control quality, the concrete was sourced locally from a ready mixed plant located in Saudi Arabia. 

The concrete beam samples were first cast in formwork and were then later cured as per the ASTM standard specification C-31 [37]. After curing, the beam surfaces were cleaned with a wet cloth and acetone, and later on were externally reinforced with CFRP strips. Epoxy (Sikadur 330) was used to attach CFRP strips to the concrete beam surface. Beam samples were then cured for three days after a careful application of CFRP over the surface of concrete using a wet layup method. Precautionary measures were adopted in order to maintain a uniform epoxy coat thickness over the concrete beam surface. 

Table 2, Table 3 and Table 4 show the material properties of the concrete, CFRP and epoxy, respectively. By using ASTM standard C-39 [38], the average compression strength of concrete was determined, while the other properties of the concrete, such as the elastic and shear moduli of elasticity, were determined using ACI 318-08 [39]. However, the material properties of CFRP and epoxy were provided by the material supplier LabsExperts, Riyadh, Saudi Arabia. 

## 5. Scale Factors

The experimental beams were scaled down using principles of modeling provided by Noor and Boswell [40]. Table 5 shows the geometric and mechanical properties of the scaled model beam in comparison to the prototype beam. The prototype beam is the full-scale beam, which is present in most residential and commercial buildings constructed in various parts of the world. The size and strength of the experimental beam samples were decided based upon the available testing facility at King Faisal University. The scale factors given in Table 5 could be applied to obtain the required forces and deformation effects of full-scale beams or prototype beams. These scale factors can also be used to obtain the forces and deformation effects at any desired scale for comparison and evaluation purposes. The steel reinforcement bars for flexure were scaled down based on the dimension of the experimental scaled beams. All of the experimental scaled beams were designed for tension-controlled failure, as per ACI 318-08 [39]. Internal reinforcement of two deformed 14 mm nominal diameter rebar was provided by experimental scaled beams, as shown in Figure 1. 

## 6. Test Setup

**Figure 4:**
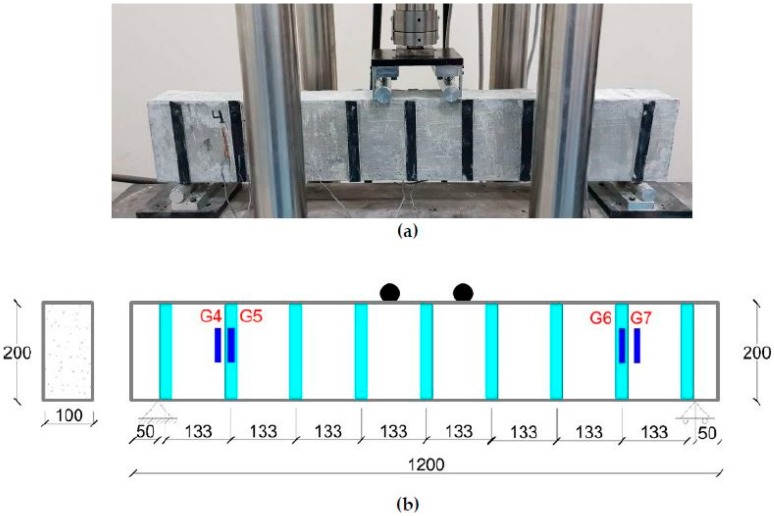
Test setup for the beam: (**a**) experimental; (**b**) schematic.

Load tests were carried out by using the bending test setup available at the highway laboratory of King Faisal University. Figure 4a,b shows the experimental and schematic test setup of the beams tested under flexural loading. The effective span of the beam was maintained at 1100 mm with the help of two roller supports, as shown in Figure 4b. Beams were tested under four-point flexural loads at mid-span under a displacement control system of 1.5 mm/min. Displacement or deflection response of the beams was measured using strain gauges placed at different locations over the concrete and CFRP surfaces. Crack pattern shapes, strains, loads and deformation values were monitored during testing for each beam, and the results are shown in the form of load-deformation curves.

## 7. Results and Discussion

### 7.1. Load Deformation Behavior

Table 6 shows all beam samples, along with their total CFRP reinforcement ratios, peak loads and mid-span deflections. All beams that were retrofitted with CFRP reinforcement showed better results than control samples in terms of peak load and mid-span deflection (ductility). B2 (F+0) shows the highest peak load value, at 141.94 kN, and the highest mid-span deflection, at 10.89 mm, among the beams.

**Figure 5:**
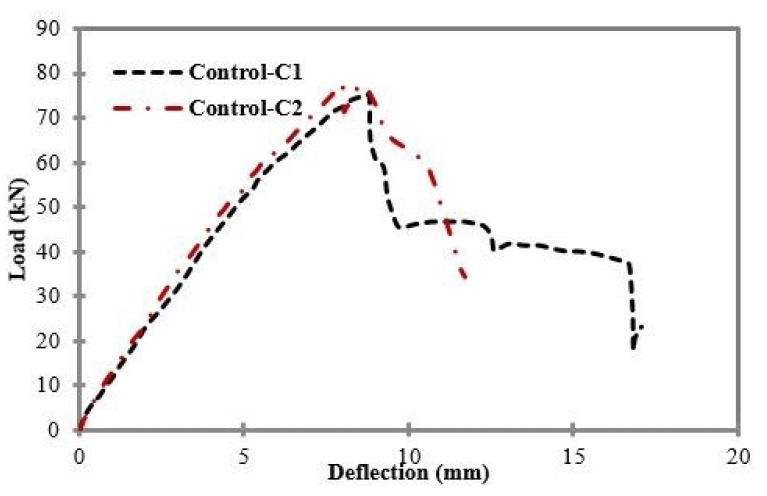
Load vs. mid-span deflection for control samples C1 and C2.

Figure 5 shows the load and deflection curves for control samples C1 and C2. Both control samples showed almost the same behavior under four-point flexural loading in terms of peak load and mid-span deflection values, i.e., 75.1 kN for control sample C1, with a mid-span deflection value of 8.78 mm; and 77.83 kN for control sample C2, with a mid-span deflection value of 7.99 mm. No external flexural and shear CFRP reinforcement were used in control samples C1 and C2. 

**Figure 6:**
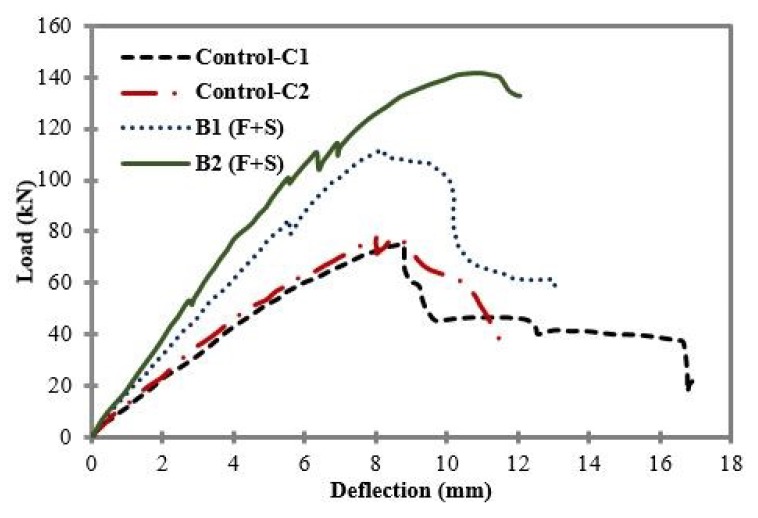
Load vs. mid-span deflection for Control samples C1 and C2, B1 and B2.

Figure 6 shows the comparison of load-deflection curves between control samples C1 and C2, and B1 and B2. The external CFRP flexural reinforcement strip width of 100 mm was kept constant in both B1 and B2. The external CRFP shear reinforcement ratio was maintained at zero in B1, while for B2 it was equal to 0.00375, i.e., half of the external flexural CFRP reinforcement ratio. B2 showed the highest peak load, at 141.94 kN, with a mid-span deflection of 10.89 mm, in comparison to B1 and the control samples C1 and C2. Beam B2 had the same flexural reinforcement as beam B1; however, B2 had more external CFRP shear reinforcement. For this reason, the strength and ductility of beam B2 were increased. Once the peak load was reached the beam sample started losing its stiffness, and the load began to decrease. 

**Figure 7:**
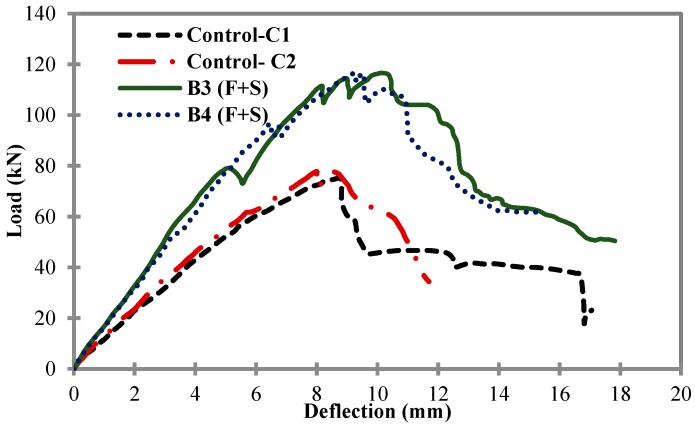
Load vs. mid-span deflection for control samples C1 and C2, B3 and B4.

Figure 7 shows the comparison of load-deflection curves between control samples C1 and C2, and B3 and B4. CFRP flexural reinforcement consisting of strips with a width of 50 mm was kept constant in B3 and B4 beams, i.e., a 0.00375 ratio. On the other hand, the external CRFP shear reinforcement ratio was equal to 0.00375 (12 strips with a width of 25 mm) in B3, while for B4 it was equal to 0.005625 (18 strips with a width of 25 mm). After the load test, B3 exhibited a peak load of 116.62 kN with a mid-span deflection of 10.17 mm, and B4 exhibited a peak load of 116.78 kN with a mid-span deflection of 9.22 mm. Both beam samples B3 and B4 showed similar behavior in terms of peak load and ductility (mid-span deflection). The addition of 6 extra external CFRP shear strips in B4 increased the peak load slightly, but did not increase the ductility of the beam much. Both beam samples B3 and B4 showed better behavior than the two control samples in terms of both peak loads and deflection values.

**Figure 8:**
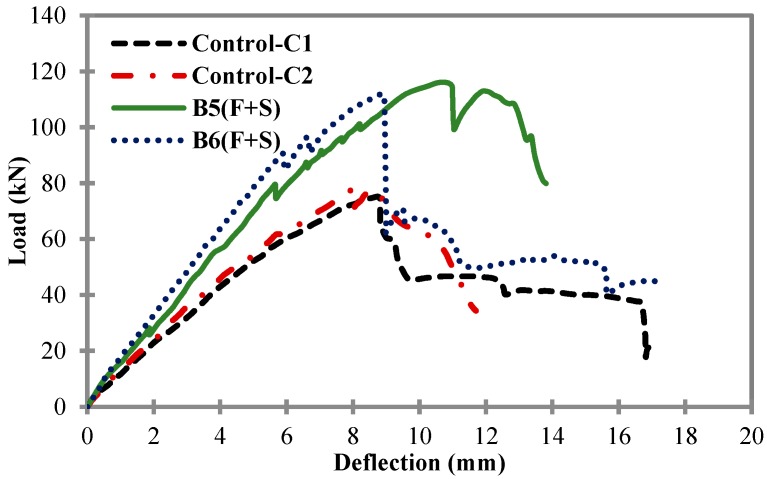
Load vs. mid-span deflection for control samples C1 and C2, and B5 and B6.

Figure 8 shows the comparison of load-deflection curves between control samples C1 and C2, and B5 and B6. After the load test, B5 showed a peak load of 116.01 kN, with a mid-span deflection of 10.56 mm; and B6 showed a peak load of 111.96 kN, with a mid-span deflection of 8.91 mm. The external CFRP flexural reinforcement was the same in beams B3, B4, B5 and B6. However, beam B5 and B6 had different CFRP external shear reinforcement ratios, of 0.003125 (10 strips with a width of 25 mm) and 0.0025 (8 strips with a width of 25 mm), respectively. Both tested beam samples B5 and B6 showed very similar results in terms of peak values and deflection, but there was a higher value of peak load in beam B5 due to the additional 2 extra external CFRP shear strips. In comparison to the control samples, both beams B5 and B6 showed a higher load-carrying capacity and improved ductility. 

### 7.2. Flexural Strain of Beams

**Figure 9:**
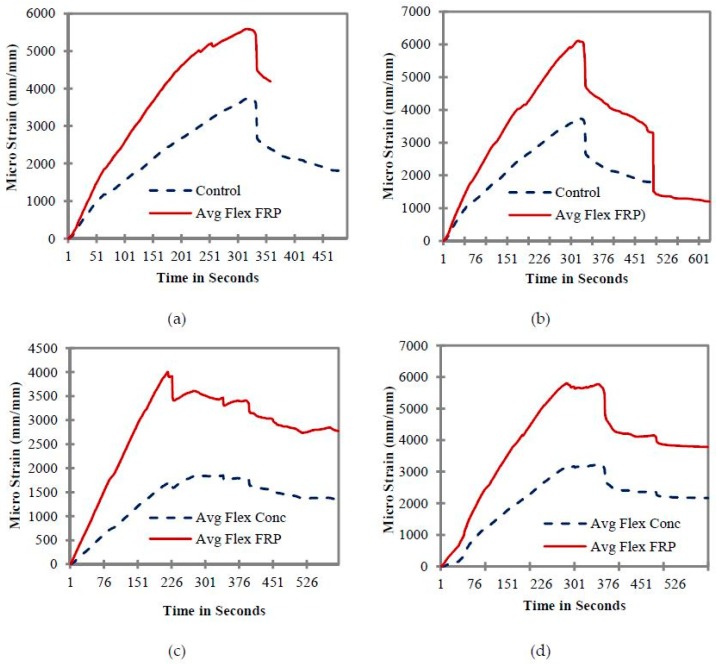

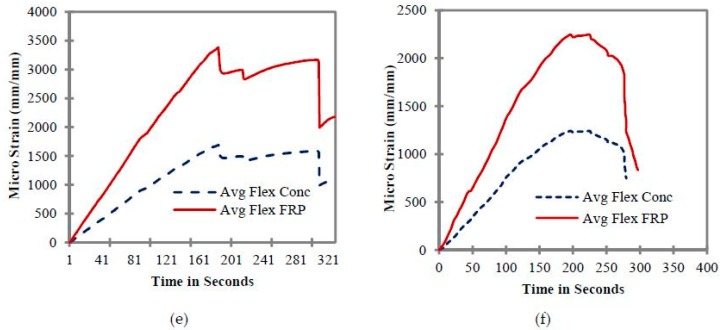
Comparison of concrete and CFRP flexural strain for (**a**) Control and B1, (**b**) Control and B2, (**c**) B3, (**d**) B4, (**e**) B5, and (**f**) B6.

The strain distributions of concrete and CFRP strips of all tested beams are shown in Figure 9. The failure modes of the CFRP-strengthened beams were found to be complex compared to the beams that do not carry any external CFRP flexural reinforcement. Strain gauges were attached at the section of maximum flexural tensile stress and provide measurements of the flexural tensile strain of the beams at extreme fiber locations. Figure 9 shows the average flexural strain values of CFRP strips in relation to average flexural strain values of concrete. The graphs of Figure 9a,b were plotted between control samples C1 and C2, and beam B1 and beam B2. The external CFRP flexural reinforcement with a strip width of 100 mm was kept constant in both beams B1 and B2. Figure 9a shows that the initial behavior of the beams remains fairly elastic until the time interval of 51 seconds; however, after this, a slight shift in the concrete and CFRP strain values was observed. This slight shift indicates a small reduction in beam stiffness and strength. Beam B1 exhibited a maximum or peak CFRP flexural strain value of 5700 micro strain and a concrete strain value of 3650 micro strain. However, in the case of CFRP, small variations in terms of rising and falling were observed (as shown in Figure 9a) prior to the peak strain values. This phenomenon could be due to detachment or debonding of some parts of the CFRP strips from the concrete surface.

Beams B1 and B2 carry the same flexural reinforcement; however, the external CRFP shear reinforcement ratio was maintained at zero in beam B1, while for beam B2, it was equal to 0.00375 (12 strips of 25 mm each). The addition of these shear strips resulted an increase in beam strength, as well as in CFRP flexural strain values, which are given in Figure 9b. Additional CFRP shear strips increased the flexural strain of the CFRP from 5700 to 6010. Fluctuation in the CFRP strain values due to slight debonding of the CFRP from the concrete surface was also observed. Moreover, the post-peak behavior (after the peak load) was significantly improved, as strain values did not suddenly decrease. In the end, the strain values of the CFRP were equal to the concrete values, showing that the CFRP had completely debonded, and its contribution was negligible. Even though beam B2 has the same external flexural reinforcement as beam B1, the addition of six extra external shear reinforcement strips with a width of 25 on at each side of the beam increased the stiffness and ductility of beam B2, thus resulting in higher strain values. Once the peak strain value was reached, the beam samples started losing their stiffness and strain value started decreasing. 

When the flexural reinforcement of the beam B3 was reduced to half that of beam B1 and B2, a significant reduction in the CFRP flexural strain values was observed (Figure 9c). Flexural behavior of the CFRP and concrete remained linear prior to the peak strain values. Once peak strain values had been reached, debonding of CFRP started, which resulted in a decrease in the strain values of CFRP and concrete. Finally, beam B3 failed due to the crushing of the concrete on the compression side.

Figure 9d shows the average flexural strain values of CFRP strips in relation to the average flexural strain values of concrete for B4. The external CFRP flexural reinforcement strips with a width of 50 mm remained the same; however, the external CRFP shear reinforcement ratio remained equal to 0.005625 (18 strips with a width of 25 mm). Beam B4 exhibited a CFRP flexural strain value of 5850 micro strain and a concrete strain value of 3120 micro strain. A small variation in the initial strain values was observed in the case of beam B4, which indicates the presence of some imperfect bond patches. The addition of shear strips improved the behavior of the beam, and it showed higher strain values when compared to beam B3. Moreover, the debonding behavior of CFRP also improved, and debonding started at a time interval much greater than that of beam B3.

Similar to beams B3 and B4, the external CFRP flexural reinforcement with a strip width of 50 mm was also kept constant in both beams B5 and beam B6; however the external CRFP shear reinforcement ratio was changed, and was equal to 0.003125 (10 strips with a width of 25mm) in beam B5, while for beam B6 it was equal to 0.0025 (8 strips with a width of 25 mm). Figure 9e,f shows the flexural strain profiles of beam B5 and B6, respectively. Both CFRP and concrete remained linear until the peak values of 3450 and 1640 had been reached. Once the peak values had been reached, a sudden drop in the strain values was witnessed, corresponding to the debonding of CFRP from the concrete surface. Beam B5 could not sustain a further increase in load and started losing its strength, as shown in Figure 9e. Beam B6 had the lowest CFRP flexural and shear reinforcement ratio among all of the beams, resulting in lower flexural strain values. Figure 9f shows a CFRP flexural strain value of 2235 and a concrete strain value of 1240, showing a higher contribution of CFRP as compared to concrete. No sudden debonding failure of the CFRP flexural strip was observed in the case of beam B6. Once the peak values had been reached, a gradual decrease in the flexural strain values of beam B6 was observed. It was also noted that beam B6 did not perform well in terms of post-peak behavior, as the strain values continued to fall upon further application of load. Reducing the CFRP external reinforcement in the case of beam B6 resulted in a drop in the flexural strain values of beam B6. To achieve the better performance of the CFRP strengthening system, beam B6 should have higher external flexural and shear reinforcement. 

### 7.3. Shear Strain of Beams

**Figure 10:**
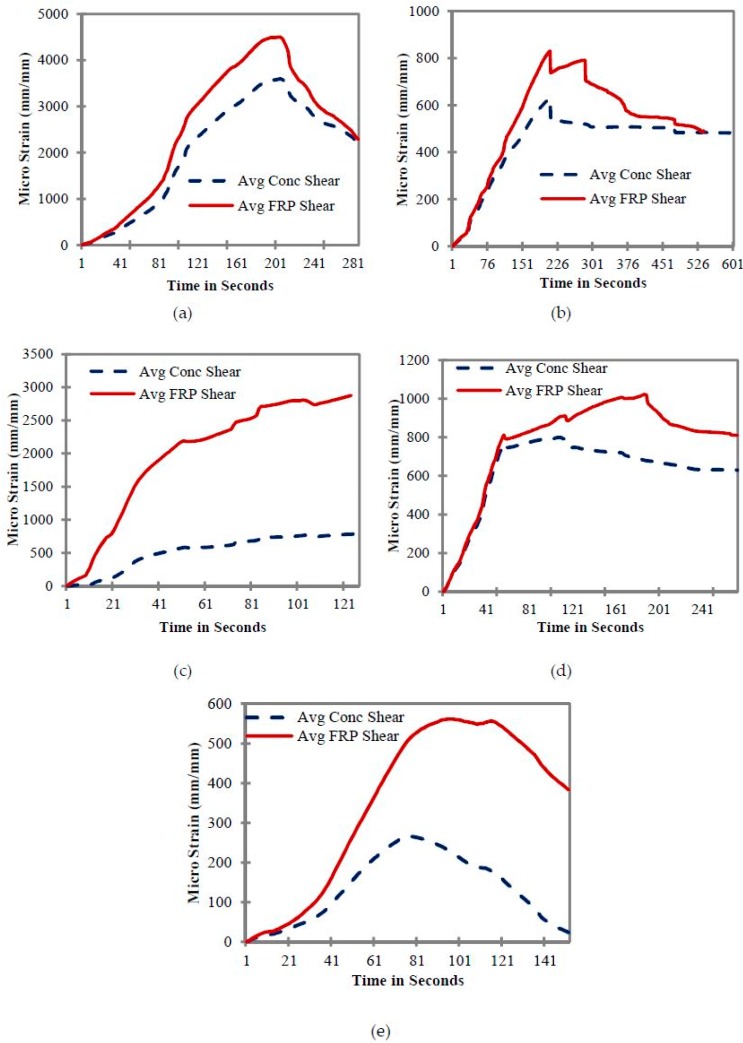
Comparison of concrete and CFRP shear strain for (**a**) B2, (**b**) B3, (**c**) B4, (**d**) B5, and (**e**) B6.

Figure 10a–e shows the shear strain values of CFRP-strengthened beam samples under flexural loading. The values reported in all these graphs are the average values of four strain gauges. The location of these gauges was planned in such a way that at each cross-section, the strain gauges carry the same shear force. Figure 10 does not show any comparison of concrete and FRP shear strain for Control C1, C2 and beam B1, as these beams do not carry any FRP shear strip on their sides (Figure 1a,b). 

Figure 10a shows the shear strain distribution of beam B2. The maximum shear strain carried by the shear CFRP strips was 4560 micro strain. However, the strain carried by the concrete was approximately 3640 micro strain which is around 1.25 times less than the strain of FRP. This shows the higher shear contribution of FRP strips compared to the concrete. The shear profiles of CFRP and concrete were gradual, and no sudden drops were observed. Initially, the values of CFRP and concrete were close, which indicates that the contribution of CFRP and concrete was very close under shear stresses. However, the gap between these values keeps on increasing until the peak value of the strain is reached, and it became evident that the contribution of CFRP in shear was significantly higher compared to the concrete.

The shear strain distribution of beam B3 is given in Figure 10b. In beam B3, lower micro strain values of both FRP and concrete were observed when compared to the beam B2. In the case of beam B3, the flexural reinforcement was reduced by one half compared to beam B2. For this reason, the flexural strength of the beam governs the overall behavior, and a higher contribution of FRP and concrete in shear could not be observed. Moreover, average shear strain values of concrete and FRP were also close to each other. Maximum average shear strain values of 821 and 609 were found for FRP and concrete, respectively. A steep drop in strain was observed at the time step of 226 s; this could be due to the formation or propagation of the flexural crack, resulting in a reduction in the effective cross section of the beam and a subsequent reduction in strain values. 

Figure 10c presents the distribution of shear strain with respect to time for beam sample B4. In this beam, the shear reinforcement of the beam increased, while keeping the flexural reinforcement same as that of beam B3. Average shear strains of 2708 and 785 were found for FRP and concrete, respectively. These values are lower than the values of sample B2, but higher than B3. This could be due to the higher contribution of FRP in shear compared to beam B3, as the shear strain values of FRP are significantly higher than the concrete strain values of beam B4. Moreover, the CFRP and concrete strain profiles show that the beam retained a higher residual capacity against shear forces; however, due to the lower flexural reinforcement of CFRP, the beam was not capable of taking any further load. 

In the case of beam B5, an overall reduction of FRP and concrete strain was observed, and the average values of FRP and the concrete strain were found to be 1011 and 792 micro strain, respectively (Figure 10d). This could be due to a reduction in the shear reinforcement of beam B5 compared to beam B4. Figure 10e presents the shear strain distribution of beam B6. A significant reduction in shear strain values was found in the case of beam B6, as it carries the minimum shear reinforcement when compared to all other beams.

In most of these cases, a sudden drop in shear strain values was not observed; a smooth or gradual increase and a decrease in the shear strain values was noted. In all beams, the shear strain values of FRP were higher, which shows a greater contribution of FRP in shear strength as compared to concrete. Moreover, the shear strain values also depend upon the bonding properties of FRP and the surface texture of concrete. Reducing flexural reinforcement resulted in a drop in shear strain values, which indicates that the full strength of FRP in shear was not fully utilized, and the flexural failure had a dominant role in governing the overall strength of the beams.

## 8. Failure Patterns

**Figure 11:**
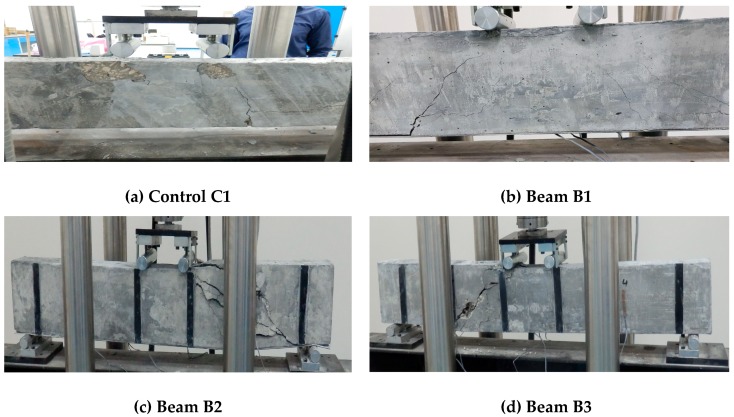

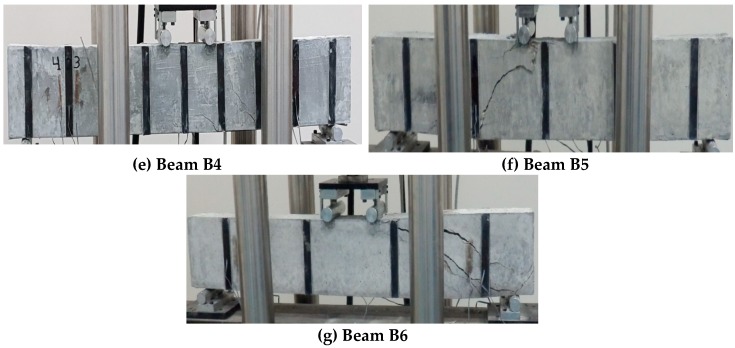
Failure pattern of the beams under flexural tests: (**a**) Control C1, (**b**) B1, (**c**) B2, (**d**) B3, (**e**) B4, (**f**) B5 and (**g**) B6.

**Figure 12:**
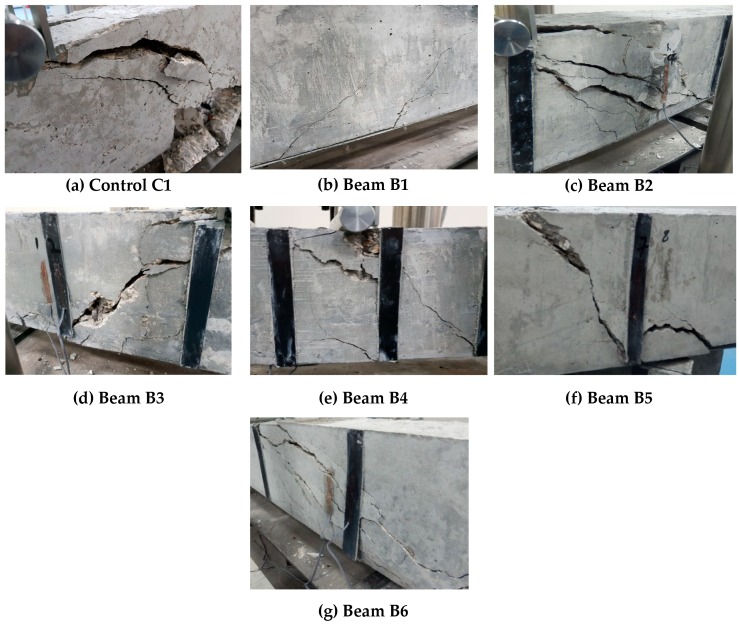
Detailed cracking pattern of the beams under flexural tests: (**a**) Control C1, (**b**) B1, (**c**) B2, (**d**) B3, (**e**) B4, (**f**) B5 and (**g**) B6.

Figure 11 and Figure 12 show the failure patterns of the tested beams. Figure 11 presents the overall cracking pattern of the beams, whereas Figure 11 gives a closer picture of the crack and failure pattern of the beams particularly at the end of each test. 

The failure behavior of the control samples is shown in Figure 11a and Figure 12a. The failure of the control beam initiated with the crushing of the concrete under the loading rollers followed by the formation of flexural crack near the mid-span of the beam as given in Figure 11a. These cracks further widened and propagated to the top of the beams with spalling of the concrete from the bottom face of the beams, as shown in Figure 12a. Flexural mode of failure was found in the control samples.

A similar kind of behavior was observed in the case of beam sample B1 (Figure 11b and Figure 12b). The failure initiated by the debonding of CFRP strip from the bottom of the concrete beam was followed by the emergence of mid-span flexural cracks (Figure 11b). With a further increase in load, the debonding between concrete and CFRP strip increased, and it started propagating from mid-span towards the support of the beam, as shown in Figure 12b. 

The failure pattern of beam B2 is shown in Figure 11c and Figure 12c. By adding strip CFRP strips to the concrete beam B2, the failure modes of the beams were changed. In beam B2, the crack initiated under the loading rollers of the machines and propagated from the compression face of the beam toward the supports as a prominent diagonal shear crack, as can be seen in Figure 11c. The presence of the shear strip hindered the movement of the crack and eventually failed and fell apart as it completely detached from the surface of the concrete beam (Figure 12c). However, the loading values and strain results showed an effective utilization of the beam, as a significant increase in load-carrying capacity was observed. 

Figure 11d and Figure 12d show the cracking pattern of the beam B3. In beam B3, a shear dominant failure like that of beam B2 was observed. However, the shear crack was steeper and remained restricted within the two FRP strips, as shown in Figure 12d. In the case of beam B4, which had the highest shear reinforcement ratio among the beams, the shear crack was steeper still. The presence of the FRP strip did not allow the crack to propagate diagonally, and eventually it moved down along the shear strip as shown in Figure 12e. The failure pattern of beam B5 was similar to that of beam B4, as shown in Figure 11e,f and Figure 12e,f, except for the formation of the wide notch at the bottom face of the beam B5. In beam B6, a diagonal shear crack over a longer length of the beam was found (as shown in Figure 11g and Figure 12g). This occurred due to its having the least shear CFRP reinforcement among all the beams, resulting in the wide spacing of the CFRP strips.

## 9. Conclusions

In the current research work, the load-deformation, flexural strain, shear strain and failure behavior of reinforced concrete beams were discussed in detail. All the beams under experiment were strengthened with different CFRP reinforcement ratios. The amount of CFRP reinforcement ratio and CFRP application layout were the main parameters of this research study. The results of the load-deformation behavior of the tested beams show that a careful selection of retrofitting scheme is vital to achieving the optimized performance of CFRP strengthened reinforced concrete members.

The research findings indicate that increasing the external CFRP shear reinforcement in beams with a weak flexural behavior is not a good choice to strengthen beams. For example, beam B4, which has a high external CFRP shear reinforcement ratio but a lower external CFRP flexural reinforcement ratio, did not show a significant increase in load-carrying capacity. On the other hand, comparison of the load-deformation behavior between beam B1 and B6 shows that a beam with a high external CFRP flexural reinforcement ratio alone may result in lower overall load-carrying capacity as compared to the other beams that carry an external shear reinforcement ratio (shear strips). Although the peak loads of the beams were different from one another, the post-peak behavior of the beams was almost similar. At higher deformation levels, the beam strengths became very close to one another.

In general, the shear strain values of the retrofitted beams were dependent on the amount of CFRP reinforcement, particularly flexural reinforcement. By decreasing the amount of external flexural reinforcement ratio in beams, a significant drop in the shear strain values was recorded. However, the addition of more side strips to the beams improved the shear behavior, and an increase in shear strain values was observed. In general, the flexural and shear strain values of CFRP were higher than the concrete strain values, showing a higher contribution of CFRP strips in the overall strength of beams.

In the absence of any CFRP external shear reinforcement, the flexural mode of failure was observed. The failure in the beams initiated due to the debonding of CFRP strips from the concrete surface. With an increase in external CFRP shear reinforcement ratio, the shear crack became steeper and remained confined within the two FRP shear strips, which shows that the beam full shear capacity was utilized. 

## Figures and Tables

**Table 1:** Preliminary testing plan.

Sr. No.	Sample Designation	Flexural Reinforcement Ratio (CFRP) *P_f_*	Shear Reinforcement Ratio (CFRP) *P_s_*	Total Reinforcement Ratio of CFRP (Flexural Shear), *P_v_*	Number of CFRP Shear Strips	Width of CFRP Flexural Strip (Mm)
1	Control-C1	N/A	N/A	N/A	N/A	N/A
2	Control-C2					
3	B1 (F+0)	0.0075	N/A	0.0075	N/A	100
4	B2 (F+S)	0.0075	0.00375	0.01125	12	100
5	B3 (F+S)	0.00375	0.00375	0.0075	12	50
6	B4 (F+S)	0.00375	0.005625	0.009375	18	50
7	B5 (F+S)	0.00375	0.003125	0.006875	10	50
8	B6 (F+S)	0.00375	0.0025	0.00625	8	50

**Table 2:** Detailed material properties of concrete and rebar.

	Material Property	Value
1.	Compressive strength of the concrete, f_c_′	28 MPa
2.	Modulus of Elasticity of concrete, E_c_	24,870 MPa
3.	Poison’s ratio of concrete, υ_c_	0.2
4.	Coefficient of Thermal expansion concrete, α_c_	9.9 × 10^−6^/°C
5.	Shear modulus of Concrete, G_c_	10,360 MPa
6.	Yield stress of steel rebar, f_y_	420 MPa
7.	Tensile strength of steel rebar, f_u_	620 MPa
9.	Modulus of Elasticity of steel, E_s_	200,000 MPa
10.	Poison’s ratio of steel, υ_s_	0.3
11.	Coefficient of Thermal expansion of steel, α_s_	11.7 × 10^−6^/°C
12.	Shear modulus of steel, G_s_	76,920 MPa

**Table 3:** Material properties of CFRP.

Material	Specific Gravity	Tensile Strength	Tensile Modulus	Bending Strength	Bending Modulus	Coefficient of Thermal Expansion	Ultimate Elongation
		(MPa)	(GPa)	(MPa)	(GPa)	(10^−6^/°C)	(%)
CFRP	1.5	1600	120	104	72	0.2	1.8

**Table 4:** Material properties of the epoxy.

Material	Specific Gravity	Tensile Strength	Tensile Shear Bond Strength	Bending Strength	Compressive Elasticity Modulus
		(MPa)	(MPa)	(MPa)	(GPa)
Epoxy	1.4	20	9.6	45	1.5

**Table 5:** Geometric and mechanical properties of the prototype and models for Beams.

		Experimental Model Value	Prototype Value	Scale Factor
**Geometry**	Length (m)	1.2	5.0	4.2
	Width (m)	0.1	0.3	3.0
	Depth (m)	0.2	0.45	2.25
**Strength**	Compressive strength (MPa)	28	20	0.95
	Shear strength (kPa)	0.78	0.76	0.97
	Density (kg/m^3^)	2400	2400	1.0

**Table 6:** Beam sample peal loads and deflections.

Sr. No.	Sample Designation	Total CFRP Reinforcement Ratio, *ρ_v_*	Peak Load (kN)	Mid-Span Deflection (mm)
1	Control-C1	N/A	75.1	8.78
2	Control-C2		77.83	7.99
3	B1 (F+0)	0.0075	111.70	8.22
4	B2 (F+S)	0.01125	141.94	10.89
5	B3 (F+S)	0.0075	116.62	10.17
6	B4 (F+S)	0.009375	116.78	9.22
7	B5 (F+S)	0.006875	116.01	10.56
8	B6 (F+S)	0.00625	111.96	8.91
